# Rapid and Highly Sensitive Detection of Ricin in Biological Fluids Using Optical Modulation Biosensing

**DOI:** 10.3390/bios15050295

**Published:** 2025-05-06

**Authors:** Eliana Levy, Linoy Golani-Zaidie, Shmuel Burg, Efi Makdasi, Ron Alcalay, Reut Falach, Ofir Schuster, Amos Danielli

**Affiliations:** 1 Faculty of Engineering, The Institute of Nanotechnology and Advanced Materials, Bar-Ilan University, Max and Anna Webb Street, Ramat Gan 5290002, Israel; ellie@uklevy.com (E.L.); linoy.golani@biu.ac.il (L.G.-Z.); burgsh@biu.ac.il (S.B.); 2 Department of Biochemistry and Molecular Genetics, Israel Institute for Biological Research, Ness Ziona 74100, Israel; efim@iibr.gov.il (E.M.); rona@iibr.gov.il (R.A.); reutf@iibr.gov.il (R.F.); 3 Department of Infectious Diseases, Israel Institute for Biological Research, Ness Ziona 74100, Israel

**Keywords:** ricin, toxicology diagnostics, optical modulation biosensing, bioterrorism, fluorescence-based immunoassay

## Abstract

Ricin, a highly toxic glycoprotein derived from the seeds of Ricinus communis, poses significant risks in bioterrorism and toxicology due to its rapid absorption and ease of dissemination. Rapid, ultra-sensitive detection is crucial for timely medical intervention and implementing security measures. However, existing methods often lack sufficient sensitivity or require lengthy processing, limiting their utility for trigger-to-treat scenarios. Here, we present an optical modulation biosensing (OMB)-based ricin assay capable of detecting low concentrations of ricin in buffer, plasma, and biological samples. The assay combines magnetic-bead-based target capture with fluorescent signal enhancement, achieving a limit of detection (LoD) of 15 pg/mL in buffer and 62 pg/mL in plasma, with a 4-log dynamic range. Optimized protocols reduced the assay time to 60 min, maintaining an LoD of 114 pg/mL in plasma while preserving accuracy and reproducibility. The assay successfully detected ricin in bronchoalveolar lavage fluid and serum from mice that were intranasally exposed to ricin, with signals persisting up to 48 h post exposure. Its rapid, high-throughput capabilities and simplified workflow make the OMB-based assay a powerful tool for toxicology, forensic analysis, and counter-bioterrorism. This study highlights the OMB platform’s potential as a sensitive and robust diagnostic tool for detecting hazardous biological agents.

## 1. Introduction

In recent years, the rapid and highly sensitive identification of biomarkers in toxicology has become vital for detecting exposure to specific compounds or materials, assessing the effects of such exposure, and identifying markers of susceptibility to toxicants [1,2]. Accurate pathogen identification serves as a critical first line of defense against bioterrorism and plays a vital role in providing life-saving treatment to potential victims [3,4]. Ricin, a highly toxic glycoprotein derived from the seeds of the castor bean plant (*Ricinus communis*), belongs to the type 2 ribosome-inactivating protein family (RIP-II toxins) [5]. It exerts its lethal effect by binding to cell surface carbohydrates, entering the cell, and inhibiting protein synthesis, ultimately leading to death [2]. Ricin consists of two distinct polypeptide chains connected by a disulfide bond [2]. The 32 kDa A chain (RTA) is an enzyme that inhibits protein synthesis by catalyzing the depuration of a single adenosine in the 28S ribosomal subunit [5]. The 34 kDa B chain (RTB) is a lectin that binds to galactose residues on the cell surface and causes endocytosis [6]. Ricin can enter the bloodstream through various exposure routes, including oral ingestion, injection, or inhalation. Among these, inhalation is the most toxic route, followed by parenteral injection. Clinical symptoms vary with the route of administration. Parenteral injection can lead to pronounced vasodilation and potentially trigger distributive shock [2]. Inhalation causes pulmonary edema and pneumonia, while injection results in localized swelling, lymph node enlargement, and hypotension, which can be fatal [2]. Regardless of the route, if left untreated, ricin poisoning can result in death within 36 to 72 h [7].

Most methods for detecting ricin have been demonstrated in buffer solution [8,9]. Some have been demonstrated in body fluids, such as plasma [10], serum [11], and abdominal fluid [12], and others have been demonstrated in drinks (milk [13,14,15], soda [16], and baby food [14]) and environmental samples (river water [17] and soil [13,17,18]). For example, Simon et al. (2015) used an enzyme-linked immunosorbent assay (ELISA) to detect 2 pg/mL of ricin in buffer within 3.25 h [13]. Feldberg et al. (2021) used mass spectroscopy to detect ricin within 150 min in human serum and clinical samples of abdominal fluid and showed a limit of detection (LoD) of 500 pg/mL [12]. Yin et al. (2012) used a bio-barcode amplification assay followed by PCR/qPCR to detect ricin in milk within 48 h, with an LoD of 0.001 pg/mL [15]. While these methods show promising results, they either lack sensitivity or their detection time is long. The main challenge to detecting ricin in human fluids is the fact that ricin is cleared from the bloodstream soon after it is introduced to the body [2,19]. Thus, to broaden the detection window and provide results promptly, rapid and highly sensitive biosensing technologies must be developed.

**Figure 1:**
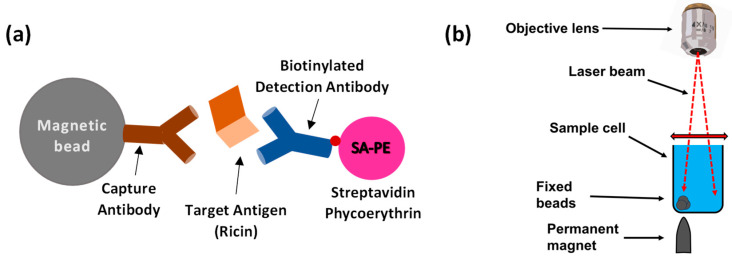
Principles of optical modulation biosensing (OMB). (**a**) In an OMB-based ricin assay, magnetic beads are coated with anti-ricin capture antibodies to bind the target antigen, ricin. Detection is achieved using a biotinylated anti-ricin antibody linked to a fluorescent marker. (**b**) A small, sharp-tipped, permanent magnet is placed beneath the sample well, causing the magnetic beads in the solution to aggregate. To eliminate background noise from unbound fluorescent analyte in the solution, the laser beam is alternated between the background solution and the fixed beads, allowing for accurate subtraction of the noise from the fluorescent signal of the beads.

Recently, we presented optical modulation biosensing (OMB), a highly sensitive and quantitative biosensing technology that can detect very low concentrations of a target analyte [20,21]. In OMB, magnetic microbeads are first conjugated to an antibody that captures the target molecule—in this case, the ricin protein. Then, a second, fluorescently labeled antibody forms a “sandwich” assay [20] with the magnetic bead and the captured protein (Figure 1a). To increase the sensitivity of the fluorescence detection, a sharp-tipped permanent magnet is placed underneath the desired well of the sample plate. This magnet concentrates the beads from the entire solution, holding them fixed at one location in the area scanned by the laser beam (Figure 1b). To reduce the need for washing steps and to shorten the detection time, the laser beam is also directed away from the bead aggregate and towards the background solution, where it measures the noise caused by unbound fluorescent molecules. By subtracting the background noise signal from the aggregate signal, the system effectively isolates the fluorescent signal of the labeled antibodies, enabling simple operation even in non-laboratory settings [20]. Previously, we demonstrated a high-throughput OMB-based platform (termed OMBi) that can automatically read a conventional 96-well plate within 10 min [20,21].

Here, we present an OMB-based ricin assay and analyze its LoD, dynamic range, and coefficient of variance (CV) in buffer and plasma. As a proof of concept, we also evaluate the clinical sensitivity of the assay using multiple samples of lung fluid and plasma collected from mice at different time points after the intranasal introduction of ricin. While we previously demonstrated the use of the OMB system for the high-throughput detection of RNA sequences in virus detection applications [20,21], this study marks the first demonstration of OMB’s capability for the rapid and high-throughput detection of proteins in complex samples in general and toxins in particular.

## 2. Materials and Methods

### 2.1. Ethical Statement

All animal procedures were conducted in accordance with Israeli regulations and received approval from the Ethics Committee for Animal Experiments at the Israel Institute for Biological Research. Animal care followed the standards set forth by the USDA Animal Welfare Act [22] and the National Institutes of Health’s Guide for the Care and Use of Laboratory Animals [23]. Female CD-1 mice (27–32 g), sourced from Charles River Laboratories Ltd. (Margate, UK), were used in this study. Prior to experimentation, mice underwent a five-day acclimation period within the animal facility. Animals were housed in filter-topped cages in a temperature- and humidity-controlled environment (21 °C; 55 ± 10% relative humidity) with a 12 h light/dark cycle simulating natural conditions. Food and water were always freely available.

### 2.2. Ricin Preparation

Crude ricin extract was prepared from *Ricinus communis* seeds following the protocol described in [24]. The seeds were homogenized in a buffer solution containing 5% acetic acid in Na_2_HPO_4_ (pH 7.4), followed by centrifugation to separate particulate matter. The resulting supernatant, containing the target toxin, underwent precipitation with ammonium sulfate at 60% saturation. After precipitation, the pellet was resuspended in phosphate-buffered saline (PBS) and dialyzed against the same buffer. Analysis of the preparation by non-reducing 10% polyacrylamide gel electrophoresis with Coomassie Blue staining revealed two predominant protein bands: one corresponding to ricin toxin (~65 kDa, approximately 80% of total protein) and another corresponding to *Ricinus communis* agglutinin (RCA, ~120 kDa, approximately 20% of total protein). Protein concentration was determined by measuring the absorbance at 280 nm using a NanoDrop spectrophotometer (Thermo Fisher Scientific, Waltham, MA, USA).

### 2.3. Optical Setup of the OMBi System

The optical setup and components of the OMB system have been described previously [20,25]. In brief, the system employs a 532 nm laser diode module (CPS532, Thorlabs, Newton, NJ, USA) emitting a 3.5 mm diameter beam at 0.5 mW (Figure S1). This beam is reshaped by plano-convex lenses (focal lengths: 200 mm and 100 mm; LA1708 and LA1509, Thorlabs), redirected by a galvanometric mirror (GVS211, Thorlabs), and deflected vertically via a dichroic mirror (BrightLine Di02-R532, Semrock, Rochester, NY, USA). Subsequently, the beam is focused through an infinity-corrected objective lens (NA 0.28, WD 34 mm; MY10X-803, Thorlabs) to form a ~150 µm spot on the bottom of a 96-well plate (Bio-Plex Pro™, Bio-Rad, Hercules, CA, USA). The fluorescently labeled targets are captured by magnetic beads, which are aggregated and immobilized at the detection site using a cylindrical magnet with a conical tip (6.35 × 25.4 mm; D4X0, K&J Magnetics, Pipersville, PA, USA), forming a ~400 µm diameter cluster. The galvanometric mirror, driven by a 2 Hz, 225 mV square wave, enables lateral scanning of the laser beam between the bead cluster and background solution. The emitted fluorescence is filtered (FF01-575/25, Semrock), redirected horizontally by a mirror (BB1-E02, Thorlabs, Newton, NJ, USA), and captured by a CMOS camera (GS3-U3-23S6M, FLIR Systems, Wilsonville, OR, USA). The 96-well plate is mounted on a motorized XY stage (ASR100B120B-E03T3, Zaber Technologies, Vancouver, BC, Canada), facilitating rapid well-to-well transitions.

### 2.4. OMB-Based Ricin Assay

To achieve optimal analytical performance using the OMBi system, we tested two configurations of capture and detection antibodies. In the first configuration, anti-ricin MH1 monoclonal antibody conjugated to magnetic beads was used as the capture antibody, and biotinylated anti-ricin MH75 monoclonal antibody was the detection antibody. The second configuration reversed the roles, with anti-ricin MH75 conjugated to magnetic beads as the capture antibody and biotinylated anti-ricin MH1 as the detection antibody. The MH1 and MH75 monoclonal antibodies, including their ricin-binding properties, were previously characterized by Noy Porat et al. [24], who provided comprehensive analytical data and binding affinity profiles for various ricin subunits. These antibodies were also successfully immobilized on a solid surface using the Octet system, a validated platform for antibody interaction analysis [26].

Here, tosylactivated M280 magnetic beads (Dynabeads M-280, #14203, Thermo Fisher Scientific, Waltham, MA, USA) were conjugated to either MH1 or MH75 anti-ricin antibodies (IIBR, Ness Ziona, Israel) following the manufacturer’s protocol [27]. Biotinylated MH1 or MH75 anti-ricin antibodies (Figure 1a) were used as detection antibodies, and streptavidin R-phycoerythrin (SA-PE) complex solution (#PJRS20-1, Agilent, Santa Clara, CA, USA) was employed as the fluorescent marker. The assay buffer comprised 0.5 g of bovine serum albumin (#A7030-50G, Merck, KGaA, Darmstadt, Germany), 5 mL of phosphate-buffered saline (PBS; PBSX10-Mg, -Ca, pH 7.4, #BP729/1LD, Hylabs, Park Tamar, Rechovot, Israel), 45 µL of deionized distilled water, and 25 µL of Tween-20 (#P1379, Merck, KGaA, Darmstadt, Germany).

### 2.5. Analytical Performance of the OMB-Based Ricin Assay in Buffer

To assess the analytical performance of the OMB-based ricin assay in buffer, we performed the following dose–response measurements. Approximately 30,000 magnetic beads conjugated with either MH1 or MH75 anti-ricin antibodies in assay buffer were added to each sample well. After removing the buffer solution, 20 µL of assay buffer containing increasing concentrations of ricin protein (0, 1 × 10^1^, 1 × 10^2^, 1 × 10^3^, 1 × 10^4^, 1 × 10^5^, and 1 × 10^6^ pg/mL) was added and incubated for 60 min at room temperature. Following a single washing step and buffer removal, the beads were incubated for 30 min with 100 µL of either biotinylated MH75 or biotinylated MH1 anti-ricin antibody, diluted to a concentration of 2 µg/µL. After a second washing step and buffer removal, the beads were incubated for 20 min with 100 µL of streptavidin–phycoerythrin (SA-PE) complex solution at a concentration of 2 µg/µL. After a single buffer replacement, the beads were analyzed using the OMBi system. During SA-PE incubation, the samples were protected from light. All incubation steps were conducted in a 96-well plate on a rotary shaker at room temperature. Each washing and buffer removal step was performed using a MagJET magnetic separation rack (Thermo Fisher Scientific, Waltham, MA, USA, Cat.# MR03). A schematic representation of the OMB-based ricin assay can be found in Figure S2. Functional validation data for the antibodies can be found in Figure S3.

### 2.6. Analytical Performance of the OMB-Based Ricin Assay in Plasma

To assess the OMBi system’s ability to rapidly detect low levels of ricin in plasma and compare its analytical performance with that in buffer, we performed a dose–response analysis of ricin protein in 50% plasma. For this, ~30,000 magnetic beads conjugated to MH1 anti-ricin antibody in assay buffer were added to each sample well. After removing the original buffer solution, 10 µL of human plasma and 10 µL of assay buffer containing increasing concentrations of ricin protein (0, 1 × 10^1^, 1 × 10^2^, 1 × 10^3^, 1 × 10^4^, 1 × 10^5^, and 1 × 10^6^ pg/mL) were added. The mixture was incubated for 60 min at room temperature, followed by a single washing step and buffer removal. The beads were then incubated for 30 min with 100 µL of biotinylated MH75 anti-ricin antibody, diluted to 2 µg/µL. After a second washing step and buffer removal, the beads were incubated for 20 min with 100 µL of SA-PE complex solution at a concentration of 2 µg/µL. Following the final buffer replacement, the beads were analyzed using the OMBi system.

Human plasma samples were purchased from Magen David Adom Blood Services (Israel) and stored at −20 °C until use. The plasma was classified and provided as “plasma for research” and was used solely as a matrix to evaluate assay performance. Since no clinical testing was performed on the plasma itself and no donor information was collected or used, IRB approval was not required.

### 2.7. Optimizing the Assay Turnaround Time

To reduce the assay turnaround time without compromising effectiveness, we performed a dose–response analysis in 50% plasma using two incubation steps (instead of three) and a shorter incubation time in each step. The incubation times of the first step were either 60 or 45 min, and the incubation times of the second step were 20 or 15 min, for total turnaround times of either 80 or 60 min. The assay included ~30,000 magnetic beads conjugated to MH1 anti-ricin antibody in assay buffer that were added to each sample well. After removing the buffer solution, we added 10 µL of plasma, 5 µL of assay buffer containing increasing concentrations of ricin protein (0, 1 × 10^1^, 1 × 10^2^, 1 × 10^3^, 1 × 10^4^, 1 × 10^5^, and 1 × 10^6^ pg/mL), and 5 µL of biotinylated MH75 anti-ricin antibody, diluted to 2 µg/µL, and incubated for 60 (or 45) minutes. After a single washing step and buffer removal, the beads were incubated for 20 (or 15) minutes with 100 µL of SA-PE complex solution at a concentration of 2 µg/µL. Following the final buffer replacement, the beads were analyzed using the OMBi system.

### 2.8. Collection of Bronchoalveolar Lavage Fluid (BALF) Samples from Mice Post-Intranasal Exposure

Bronchoalveolar lavage fluid (BALF) samples were collected from 20 mice at the Israel Institute of Biological Research, Israel. Five samples were obtained from control mice that were not exposed to ricin. The remaining 15 mice were anesthetized via the intraperitoneal injection of ketamine (1.9 mg/mouse) and xylazine (0.19 mg/mouse), and intranasally intoxicated with crude ricin at a dose of 9.6 µg/kg (~2LD_50_) [28], administered as 25 µL per nostril. These mice were sacrificed at 6, 24, and 48 h post exposure (five mice at each time point), and BALF was collected by flushing the lungs with 1 mL of PBS using a tracheal cannula. The BALF samples were centrifuged at 950× *g* for 10 min at 4 °C. Supernatants (50 µL each) were stored at −20 °C and thawed prior to the OMB assay. The assay was conducted as described in Section 2.5.

### 2.9. Collection of Serum Samples from Mice Post-Intranasal Exposure

Serum samples were collected from four mice at the Israel Institute of Biological Research, Israel. One sample was obtained from a control mouse not exposed to ricin. The remaining three mice were anesthetized via intraperitoneal injection of ketamine (1.9 mg/mouse) and xylazine (0.19 mg/mouse), and intranasally intoxicated with crude ricin at a dose of 9.6 µg/kg (~2LD_50_), administered as 25 µL per nostril. These mice were sequentially sacrificed at 24, 48, and 72 h post exposure, and their serum was collected. All samples (30–50 µL each) were stored at −20 °C and thawed prior to the OMB assay. The assay was performed as described in Section 2.5.

### 2.10. Data Analysis

For each concentration in the dose–response experiments, the mean and standard error of the mean were calculated along with the mean (${µ}_{blank})$ and the standard deviation ($\sigma_{blank}$) of the blank measurements. The Limit of Blank (LoB) was calculated as follows [29]:

(1) $$LoB={µ}_{blank}+1.645\times \sigma_{blank}.$$

The LoD was then determined using a pooled standard deviation of low-concentration measurements ($\sigma_{pooled}$), calculated as [29]

(2) $$LoD=LoB+1.645\times \sigma_{pooled}.$$

The CV was computed by dividing the standard deviation by the mean value of each concentration. The dynamic range of each dose–response was defined as the ratio between the highest and lowest detectable concentrations.

## 3. Results

### 3.1. Analytical Performance of OMB-Based Ricin Assay in Buffer

**Figure 2:**
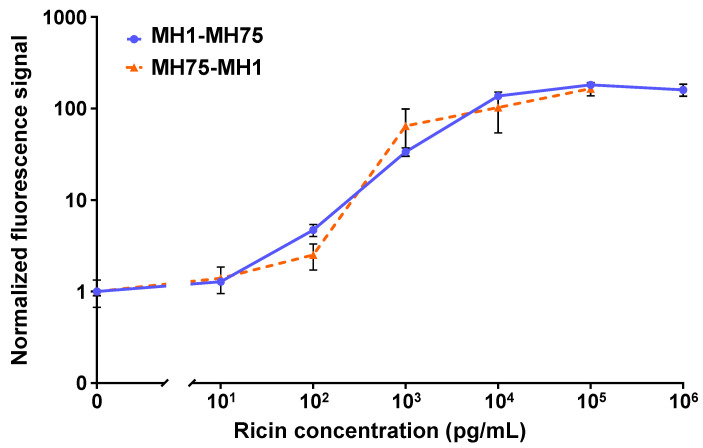
The analytical performance of the OMB-based ricin assays in buffer. For the MH1-MH75 configuration (solid blue line), the dynamic range was 4-log, the LoD was 15 pg/mL, and the CV was less than 15%. For the MH75-MH1 configuration (dashed orange line), the dynamic range was 4-log, the LoD was 100 pg/mL, and the CV was less than 53%. For the MH1-MH75 dose–response curve, the error bars represent the standard error of five experiments (n=5), with six repetitions at the blank concentration and three repetitions at each of the other concentrations in each experiment. For the MH75-MH1 dose–response curve, the error bars represent the standard deviation of 17 repetitions at the blank concentration (n=17) and 5 repetitions at each of the other concentrations (n=5).

Figure 2 shows the dose–response curves for the two configurations tested in the OMB-based ricin assay. For the MH1-MH75 configuration, the dynamic range was 4 logs, with an LoD of 15 pg/mL and a CV of less than 15%. For the MH75-MH1 configuration, the dynamic range was 4-log, the LoD was 100 pg/mL, and the CV was less than 53%. Based on these results, the optimal antibody configuration selected utilized anti-ricin MH1 as the capture antibody and biotinylated anti-ricin MH75 as the detection antibody.

### 3.2. Analytical Performance and Turnaround Time Optimization of the OMB-Based Ricin Assay in Plasma

**Figure 3:**
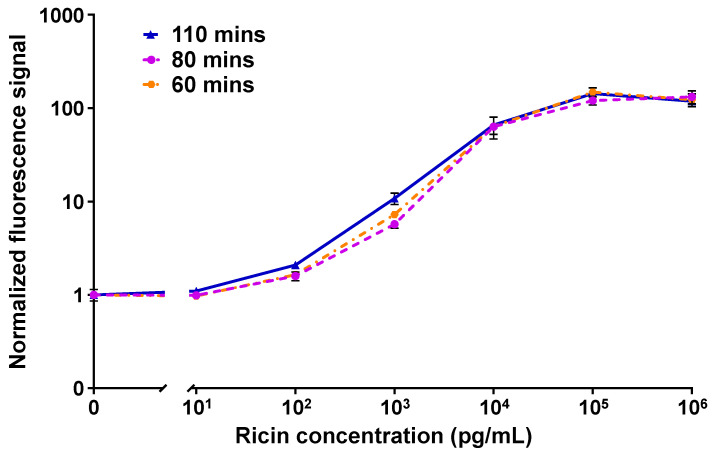
The turnaround time optimization results for the OMB-based MH1-MH75 ricin assay in 50% plasma. In a series of experiments, the turnaround time was reduced from 110 min (solid blue line) to 80 min (dashed purple line), and then to 60 min (dashed-dotted orange line). The error bars represent the standard error of three to five experiments (n=3–5). The calculated LoDs of the 110, 80, and 60 min assays were 62 pg/mL, 128 pg/mL, and 114 pg/mL. For each experiment, the mean signal of the blank concentration was calculated based on six repetitions, while three to four repetitions were performed for all other concentrations.

Figure 3 shows the dose–responses of the MH1-MH75 configuration of the OMB-based ricin assay in 50% plasma with different turnaround times. The calculated LoDs of the 110, 80, and 60 min assays were 62 pg/mL, 128 pg/mL, and 114 pg/mL; the CVs were less than 21%, 16%, and 26%; and the dynamic range was 4-log.

### 3.3. Detecting Ricin in Bronchoalveolar Lavage Fluid and the Serum of Mice Post-Intranasal Exposure to Ricin

**Figure 4:**
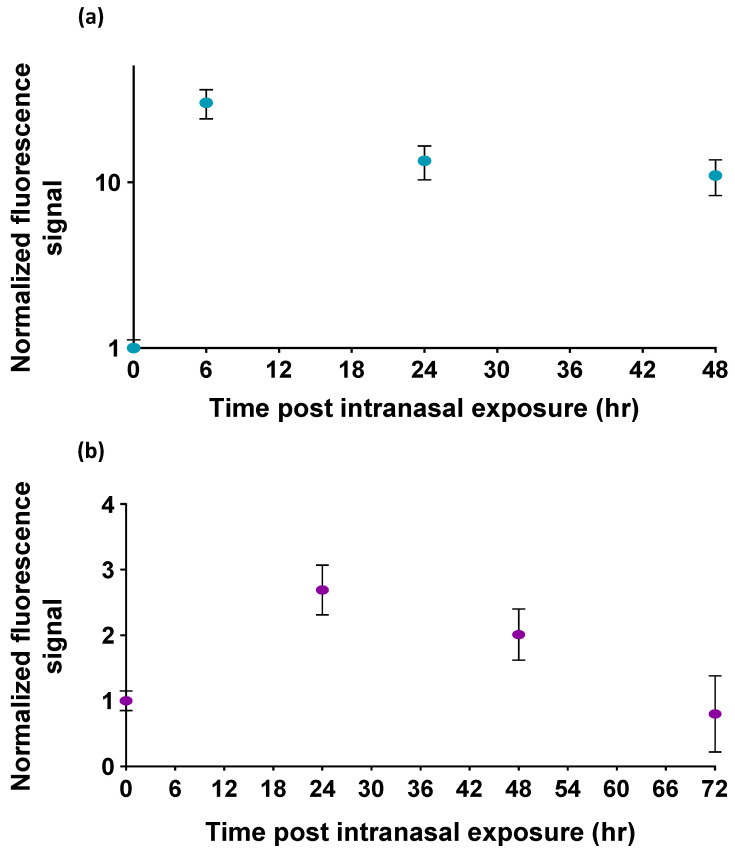
The detection of ricin in the bronchoalveolar lavage fluid (BALF) and serum of mice following intranasal exposure. Normalized fluorescence signals in (**a**) BALF samples and (**b**) serum samples of mice after intranasal exposure to ricin. The highest detected ricin levels in murine BALF samples were observed at 6 h, and a detectable signal remained in samples even at 48 h. Serum samples from mice showed the highest signal at 24 h, with detectable levels persisting up to 48 h after exposure. The signal at 72 h was not significantly different from the control. The error bars of the BALF sample experiment represent the standard deviation of five mice (n=5) at each time point. The error bars of the serum sample experiment represent the standard deviation of four to seven repetitions for each time point $\left(n=4\;–\;7\right)$.

The normalized fluorescence signals of the OMB-based ricin assay for bronchoalveolar lavage fluid samples are shown in Figure 4a. The fluorescence signals detected at 6, 24, and 48 h post exposure are approximately 10–30 times higher than the signals observed in mice not exposed to ricin. Similarly, the normalized fluorescence signals for serum samples are presented in Figure 4b. The fluorescence signals detected at 24 and 48 h post exposure are significantly higher—2.4 and 2.0 times, respectively—than the signal from non-exposed mice (*p*-value < 0.00015). However, the fluorescence signal detected at 72 h post exposure is not significantly different from the signal observed in non-exposed mice.

## 4. Discussion

Current methods for detecting ricin in body fluids are challenged by their lack of sensitivity or extended detection time. Moreover, most methods have only been demonstrated in buffer solutions, beverages, and environmental samples, with few studies evaluating their performance in body fluids (Table 1). Here, we present a new highly sensitive and rapid OMB-based assay for ricin detection in various biological matrices. The OMB-based ricin assay achieved an LoD of 15 pg/mL in buffer and 62 pg/mL in plasma. These LoD values are competitive with, and often superior to, existing methods, such as mass spectrometry and bio-barcode amplification assays, which either require extended time-to-results or specialized laboratory environments. The OMB-based assay’s 4-log dynamic range and consistent CV of less than 21% across experiments further highlight its robustness and reproducibility, demonstrating its versatility for both clinical and environmental sample analysis. The assay’s enhanced sensitivity arises from a combination of three factors: (1) the use of a sharp-tipped permanent magnet that concentrates beads within the laser beam’s detection zone; (2) the OMB system’s ability to optically isolate the signal from unbound fluorophores via modulation; and (3) optimized antibody pairs with high affinity and specificity for ricin.

Reducing the assay turnaround time is crucial in clinical and field settings. Here, we showed that a 60 min assay achieved an LoD of 114 pg/mL in plasma, with only a modest decrease in sensitivity compared to the 110 min protocol. This optimized protocol maintains an acceptable CV (<26%) and retains the 4-log dynamic range, making it suitable for time-sensitive scenarios, such as emergency responses to potential bioterrorism. This trade-off between speed and sensitivity emphasizes the adaptability of the OMB assay to different operational needs. For high-stakes applications, where rapid decision making is critical, the 60 min protocol offers a practical solution without significantly compromising performance.

The ability to detect ricin in murine BALF and serum samples following intranasal exposure demonstrates the potential clinical relevance of the OMB assay. The highest fluorescence signals in BALF were observed at 6 h post exposure, with detectable levels persisting up to 48 h. The signal-to-noise ratio ranged between 10 and 30. Similarly, serum samples exhibited peak detection at 24 h, with significant signals observed up to 48 h. However, the signal-to-noise ratio in serum was only between 2 and 2.4. By 72 h post exposure, ricin levels in serum were indistinguishable from controls. These results align with the known pharmacokinetics of ricin, where the toxin is rapidly cleared [19]. The OMB assay’s sensitivity within this narrow detection window is a critical advantage, enabling an effective identification of exposure in scenarios where time is a limiting factor. Additionally, the differential detection dynamics between BALF and serum underscore the importance of sample type in optimizing diagnostic accuracy.

The OMB platform offers several key advantages over traditional detection methods. First, the use of magnetic beads and optical modulation effectively reduces background noise, enhancing sensitivity and reliability. Second, its simplified workflow, with fewer washing steps and shorter turnaround times, facilitates its use in resource-limited or non-laboratory settings. Third, the high-throughput capability of the OMBi system enables simultaneous analysis of multiple samples, making it ideal for large-scale screening efforts. Furthermore, its compact design and minimal instrumentation requirements position OMB technology as a promising tool for point-of-care diagnostics. Additionally, we detected low concentrations of ricin up to 48 h post exposure. These attributes are particularly relevant for emergency response teams managing bioterrorism threats or outbreaks of ricin poisoning.

Despite its promising performance, the OMB-based ricin assay has certain limitations. Due to ricin’s rapid clearance from the body, detecting it beyond 72 h post exposure remains challenging. Future efforts should focus on enhancing the assay’s ability to detect ricin metabolites or immune responses to extend the detection window. Additionally, while the current study demonstrated the assay’s utility in mouse models, further validation in other sample types is necessary to establish its clinical applicability. Another area for improvement is scalability for field deployment. Integrating the OMB assay with portable and automated detection systems could enhance its usability in diverse settings, including remote locations or disaster zones, and could minimize the risk of human error. Developing multiplexed OMB platforms capable of detecting multiple biotoxins simultaneously could further expand its applications in comprehensive threat detection.

The rapid and sensitive detection of ricin presented in this study addresses critical needs in toxicology and bioterrorism preparedness. By providing a reliable tool for early detection, the OMB-based assay has the potential to mitigate the impact of ricin exposure through timely medical intervention and containment measures. Its adaptability to non-laboratory environments and its high-throughput screening capabilities make it an invaluable addition to the arsenal of diagnostic technologies for biodefense.

## 5. Conclusions

In conclusion, the OMB-based ricin assay offers a powerful combination of high sensitivity, rapid detection, and operational simplicity, making it a robust and reliable solution for identifying ricin in both clinical and environmental samples. The assay successfully detected ricin in biological fluids from mice exposed to ~2 LD_50_ of ricin, demonstrating sufficient sensitivity for identifying toxin presence even at sublethal doses and at delayed time points—conditions under which conventional assays often fail. This is especially critical given ricin’s rapid clearance from circulation and the narrow diagnostic window following exposure. Furthermore, while this study focused on ricin, the OMB platform’s modular antibody-based design can be readily adapted to detect other protein-based toxins, such as botulinum toxin or staphylococcal enterotoxin, through appropriate antibody selection. With ongoing development and validation, the assay’s performance can be further optimized, extending its application potential and solidifying its role in critical diagnostic and emergency response frameworks.

## Figures and Tables

**Table 1:** Comparison of current ricin detection methods.

Detection Method	Limit of Detection [pg/mL]	Assay Time [Hours]	Matrix	Reference
Optical-based methods				
OMB-based ricin assay	114	1	Plasma (50%)	This paper
Electro-chemiluminescent assay	50	2.5	Nasal swab washing buffer, soda, sugar, milk, and talc (5% *w*/*v* in PBS)	Guglielmo-Viret et al. (2007) [16]
Mass spectrometry	1000	3	Environmental samples (soil/asphalt/vegetation)	Feldberg et al. (2020) [18]
Mass spectrometry	5000	2.5	Human serum (50%) and real-life clinical sample of abdominal fluid (collected 72 h post injection)	Feldberg et al. (2021) [12]
Dual-readout immunosensor (fluorescence and electrochemiluminescence)	5.5	1	Buffer, environmental samples (river water/soil/tap water)	Feng et al. (2022) [9]
SERS-based sandwich immunoassay	1000 (buffer) 5000 (plasma)	4	Buffer, plasma (10%)	Wang et al. (2023) [10]
Nanoparticle-based methods				
Bio-barcode amplification assay followed by PCR/qPCR	0.001	2.5	A mixture of milk and water	Yin et al. (2012) [15]
Colorimetric biosensor	20,000	1	Pepsi and milk powder diluted in phosphate-buffered saline (PBS)	Hu et al. (2014) [30]
TMR biosensor	1000	0.25	River water, fertilized soil, butter biscuit, rabbit blood	Mu et al. (2019) [17]
ELISAs				
Colorimetric and chemiluminescent ELISA	100	5	Assay buffer, human urine (10%), human serum (2%)	Poli et al. (1994) [11]
ELISA	2	3	Buffer, milk, soil	Simon et al. (2015) [13]
Cell-based assays				
Cell-based assay	600	~24	Neutralized disinfectant samples	Rastogi et al. (2010) [31]
Cytotoxicity assay	5600	24	Carrot juice, baby food, milk	Pauly et al. (2012) [14]

## Data Availability

Data will be made available on request.

## Supplementary Materials

The following supporting information can be downloaded at https://www.mdpi.com/article/10.3390/bios15050295/s1, Figure S1: 2D schematic of the OMB optical setup; Figure S2: Schematic representation of the OMB-based ricin assay; Figure S3: Functional validation of antibody conjugation to magnetic beads.

## Abbreviations

The following abbreviations are used in this manuscript:

OMB	Optical modulation biosensing
LoD	Limit of detection
BALF	Bronchoalveolar lavage fluid
CV	Coefficient of variance
