# Supplementary material for: Rapid and Highly Sensitive Detection of Ricin in Biological Fluids Using Optical Modulation Biosensing
Source: Biosensors (Basel). 2025 May 6;15(5):295. doi: 10.3390/bios15050295 (PMC12109947; doi:10.3390/bios15050295)
Supplement: Supplementary file 1 [file biosensors-15-00295-s001.zip › biosensors-3562297-supplementary.pdf]

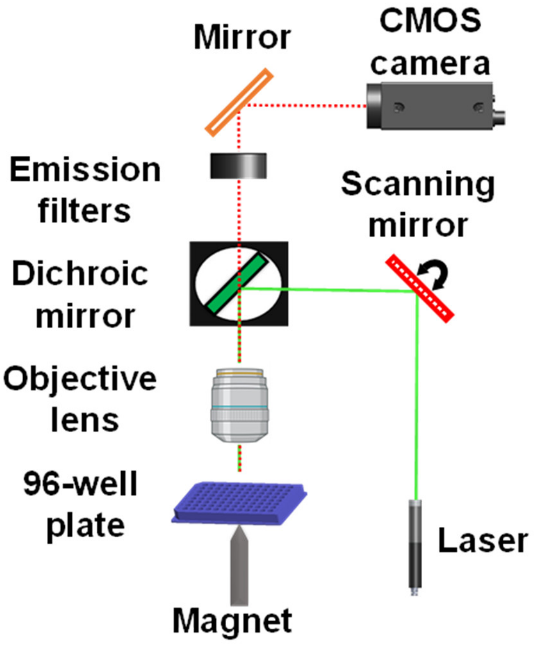

Figure S1: Two-dimensional schematic of the OMB optical setup. A 532 nm laser diode emits a 3.5 mm diameter, 0.5 mW beam, which is reshaped by plano-convex lenses (focal lengths: 200 mm and 100 mm; not shown), redirected by a galvanometric mirror, deflected vertically via a dichroic mirror, and focused through an infinity-corrected objective lens to form a ~150 μm spot on a 96-well plate. The fluorescently labeled targets are captured by magnetic beads which are aggregated at the detection spot using a cylindrical magnet with a conical tip, forming a ~400 μm cluster. The galvanometric mirror, driven by a 2 Hz, 225 mV square wave, enables lateral scanning of the laser beam between the bead cluster and background solution. The emitted fluorescence is filtered, redirected horizontally by a mirror, and captured by a CMOS camera. A motorized XY stage facilitates rapid well-to-well transitions.

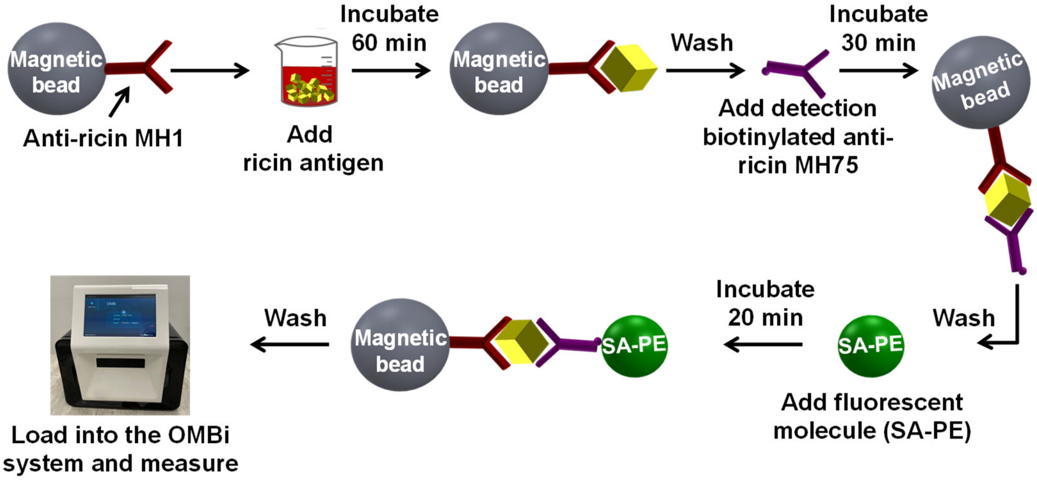

Figure S2: A schematic representation of the OMB-based ricin assay.

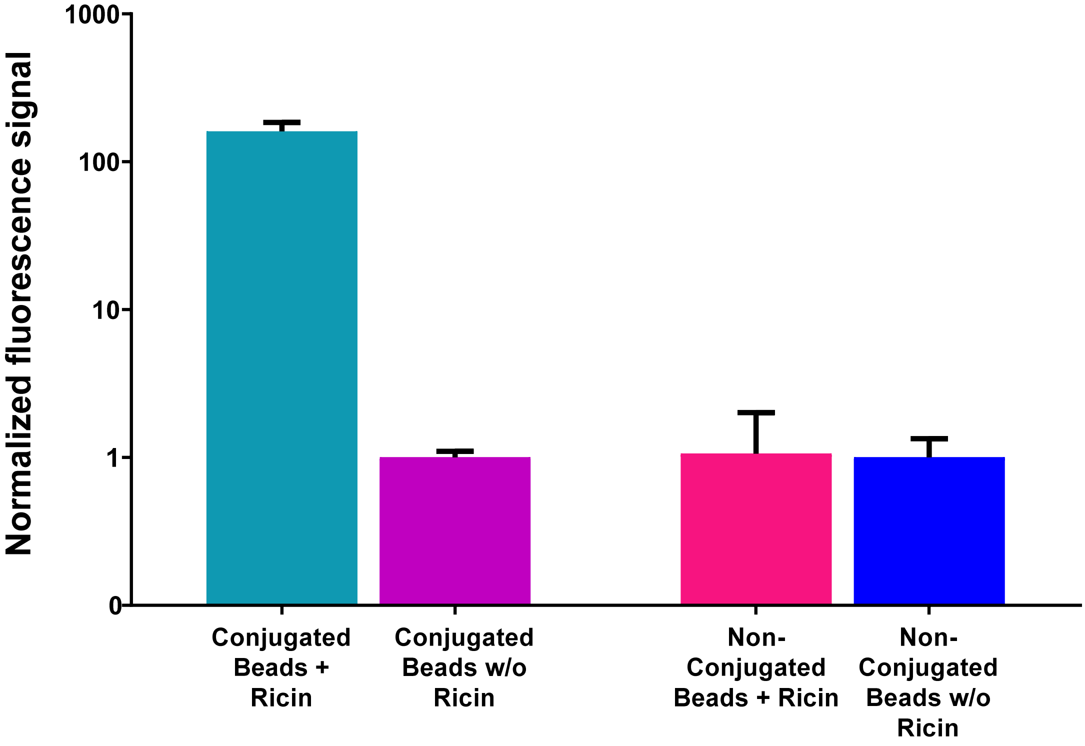

Figure S3: Functional validation of the antibody conjugation to magnetic beads. We experimentally validated the conjugation of MH1 capture antibodies to magnetic beads using the OMB-based ricin assay. Beads either conjugated with MH1 antibodies or left unconjugated (naked tosyl-activated beads) were tested in the presence and absence of ricin. Beads conjugated with MH1 antibodies produced a significant signal increase in the presence of ricin compared to the blank (no ricin), while the naked beads showed no signal difference between the ricin and blank samples. All signals were normalized to the blank sample with conjugated beads (no ricin). Error bars represent the standard deviation of three independent experiments ($n = 3$).
